# Surgery of the Kidney1Read at the Buffalo Academy of Medicine, November 1, 1898.

**Published:** 1899-01

**Authors:** Marcell Hartwig

**Affiliations:** Buffalo, N. Y.


### BUFFALO MEDICAL JOURNAL.

VOL. XXXVIII. JANUARY, 1899. No. 6.

### Original Communications.

### SURGERY OF THE KIDNEY.[1]

BY MARCELL HARTWIG, M. D., Buffalo, N. Y.

HAVING given a promise, I have to fulfill it, though I step before you with lack of confidence of interesting you, inasmuch as I have nothing new to proffer. Once in a while making a halt and surveying a field of work accomplished in medicine is worthy of time spent, if not so fascinating as novelties.

In bygone times there was of renal surgery very little, if we except paranephritic abscesses, and not until Simon, of Heidelberg, published his foundation work, *Chirurgie der Nieren*, in 1871, was there enough deserving a paper of our title. Today we can speak of eleven affections demanding frequent surgical interference with the kidney or its close neighborhood ; (1) Wounds and bruises ; (2) paranephritis ; (3) pyelitis ; (4) stone in the kidney ; (5) hydronephrosis ; (6) cystic degeneration ; (7) tuberculosis ; (8) tumors—monocysts and echinococcus included ; (9) floating kidney ; (10) ureteritis ; (11) nephralgia.

I presume you do not expect the rudiments of surgery in my paper, therefore I shall not dwell upon such, but if I mention one thing or another well known to many, it may not be so to some, and I hope to be excused. Even to those who are well versed with the surgery of the kidney some things may serve to refresh the memory.

*Traumatic Bleeding.*—The latest article about this subject by Nasse, who was recently killed in the Alps, read before the Berlin Medical Society, has a careful record of the literature and states that Studsgaard was the first to make nephrectomy for a tear in the

1. Read at the Buffalo Academy of Medicine, November 1, 1898.

kidney in 1889. Everybody will subscribe today to Nasse's general ideas: that where death by hemorrhage is threatened, the bleeding must be attacked locally by stitching, tampons and the like, and where the chief bloodvessels, veins or arteries are torn, by nephrectomy; if only branches, we may succeed with Peyrot by applying temporary clamping of the bloodvessels. The difficulty lies in the diagnosis from shock. Here only general principles can guide. In cases of doubt an exploratory incision is admissible; where a hematoma is not forming, the incision should be abdominal; where a hematoma exists, lumbar. Wounds which we make ourselves in the substance of the kidney should be aid in the line habitual in post mortem exploration, as there are the smallest bloodvessels and stitched afterward through the whole substance. It is not to be forgotten that too strong tying would cut the tissue of the organ and that other than absorbable ligatures could cause later formation of stone. Whether intensely hot applications, 115° to 120° or more, will do without precluding primary union is not established yet. Where primary union and stitching, for example in bullet wounds, are excluded, I deem cautious trials admissible.

*Paranephritis* we call (in distinction of perinephritis, which is an inflammation of the capsula propria and which has little surgical interest,) the inflammation of the fatty capsule. There is a secondary fibrous form which has little surgical interest, except that it makes the enucleation of a kidney very difficult, while the usual is the purulent form. This it seems is (excluding wounds) almost always due to spontaneous perforations of the kidney or its pelvis. Yet metastatic abscess can happen; abscess from contiguous inflammation, originating at the appendix, from caries and intestinal perforation is by no means rare; but properly it ought not to be called paranephritis.

Finally, there are cryptogenetic cases, *id est*, where the original cause remains unknown. The symptoms are pain, puffiness, edema; finally, redness and, of course, fever. Exceptionally it imitates coxitis by contracture of the iliopsoas and if extension is great, even pleuritis can really develop from it. The treatment is, of course, incision and drainage every time. A number of cases which I saw healed promptly; one I lost, and it seemed to me from absorption of bichloride, as without bleeding death followed too soon. Unless great putrefaction is present, syringing is unnecessary, as in purulent pleurisy: we may at least choose less poisonous chemicals for lavage, say $\frac{1}{3}$ per cent. salicylic acid or thymol. I

am sorry I could not obtain a post mortem; so the case remained "cryptogenetic."

*Pyelitis*, respectively pyelonephritis.—This is practically the most important and most thankful surgical kidney. Not unlikely before Simon some pyelitis cases were drained like abscesses. They are very frequently conjoined, or caused by stone in the pelvis of the kidney, so we can consider them together. Not until Rovsing proved that injection of certain germs into the ear-vein of rabbits produces pyelitis when the ureter is tied, did we positively know that a pyelitis hematogenes descendens exists. Bradford, in London, obtained lately pus in the pelvis by tying the ureter in dogs three times out of twelve; but as he cut between two ligatures his experiments (which showed atrophy of the kidney to follow) are not conclusive as infection cannot be excluded. Posner has proved that if intestine and ureter are ligated without local infection, bacterium coli enters the circulation and urine and that this may be the modus operandi how calculus in the kidney may produce pyelitis. Nature, though, shows conclusively that germs from the blood can locate in the kidney by the fact that tuberculosis occasionally occurs in the kidney. The excretory action of the kidneys being so lively we should rather wonder that descending pyelitis does not oftener occur. Possibly the future will show such origin for the non-surgical kidney diseases. The ascending pyelitis is probably not as frequent as we ought to expect, because of a valve-like action of the ureteral papillæ and of a descending trend of the ureteral peristalsis.

*Diagnosis from Cystitis.*—Dr. George Rosenfeld, in Breslau, comes to the conclusion (*Berlin Klin. Wochenschrift*, July 25, 1898,) that alkaline reaction does not occur in uncomplicated pyelitis, that the most intense cystitis hardly ever shows more than 0.1 per cent. of albumin; that if all the pus cells are stellated it seems pyelitis; that if the red-blood corpuscles are for the most part disintegrated and the amount only microscopical, pyelitis is probable, provided there is no tumor of the bladder. Characteristic is the great amount of albumin in comparison with the number of leucocytes. According to Goldberg, in Cologne, pus in nephritis is mononuclear; in pyelitis, polynuclear. He further states that if pyelonephritis is absent, even 5,000 leucocytes to the c.cm. urine furnish only one per mille albumin. He counted as high as 54 milliards of leucocytes which are emptied in the urine in twenty-four hours. Far more than a man has.

Thus we may fancy how Landau saw one hydronephrosis turn to pyelitis. Müller, of Oldenburg, in 1880, describes a case of hydronephrosis where after he had established a fistula, the fistula closed; after a while the sac refilled, burst and caused paranephritis.

*Treatment of Pyelitis.*—Calculosa and without calculus has been hitherto considered as *toto cœlo* different, but we will see that this idea is wrong today, and that both are surgical kidneys, as well as calculus without pyelitis. I will admit that some light forms (catarrhal) of pyelitis may get well by giving arbutin, chlorate of potash, turpentine, urotropine, and the like, conjoined with plentiful drinking of mineral waters, but I have not seen a single perfect recovery in twenty-five years of my practice. Flaischlen removed a pyonephritis which had lasted a quarter of a century. The reason seems to me very clear. All purulent inflammations are practically due to cocci or bacteria and such multiply indefinitely in mucous membranes, only too readily if detained the least in their escape; although, of course, the different kinds will show more or less resistance. A simple uncomplicated pyelitis nobody will consider a surgical disease, but after all medical means are exhausted it becomes such. I doubt not that in future we will drain such cases. I could find only one in the literature which Hahn cured by twenty-four days' drainage. A cause was not found. The germ in the case was established by Hirschlaff as the bacterium lactis aerogenes, Escherich's, in pure culture. Where a hindrance to the escape of pus exists it must be removed. Where, as most frequently, it appears as a calculus, we of course remove it. Everybody does. But what of strictures in the ureter? Formerly they were considered cause for nephrectomy. Today we ought to dilate from above or below. Conservative surgery of the kidney is the watchword today. Since the invention of the cystoscope by Nitze and Casper, ureteral catheters as an addition, and since Kelly has extended *my* method of viewing the bladder as a whole in women and proven its applicability in man, as I predicted in my article in the *Medical News*, August 13, 1892, we can more readily drain from below than Simon, who has already done so (in woman) for diagnostic as well as for therapeutic purposes.

I show you here Simon's original ureteral catheter. There are not many cases on record yet and I shall cite you some to show that success is possible. In principle though, in the majority of cases, we ought to drain from above—from an incision into the pelvis of the kidney—as such can be maintained indefinitely and as it is the only way which admits the finger and thus insures that we do not overlook a calculus.

The above-mentioned cases, as far as I found in the time I could devote to search, are : Bozeman cured two cases by introducing the ureteral catheter from a vesicovaginal fistula and syringing one half per mille bichloride of mercury into the renal pelvis. Pawlik had several cases. Nitze expects more from injecting nitrate of silver. *Bureau du Traitement Chirurgical des Pyonephroses Thèse* de Paris, 1890, contains a case. Casper reported, 1895, two cases of washing the pelvis per vias naturales in pyelitis gonorrhoica. In the first case the catheter remained three days in the kidney and he made daily instillations of 1 to 2 per cent. silver. In the second case he catheterised the ureters six times. In both cases all other means previously employed had failed. L. Bazet, of San Francisco, whose paper appeared in the *Philadephia Medical Journal*, October 22, 1898, just came to my hands through the kindness of Dr. Schröter, takes the same stand which I am just advocating. He says, " In cases of pyelonephritis without distension nephrotomy intervenes as a precious resource to secure by a double road the drainage and disinfection of the pelvis, when catheterisation and disinfection per vias naturales proves futile."

The modern treatment for pyelitis therefore will remain after medical means fail, incision and drainage ; if this fails, nephrectomy. Some have favored primary nephrectomy, because the secondary, when the fatty capsule has changed into fibrous tissue and intimate adhesions have formed, is more difficult of execution ; but the danger of nephrectomy is certainly greater than of nephrotomy ; and many a kidney which seemed doomed has resumed its work entirely or partially at least. Further, we find after nephrotomy, by observing the urine, additional evidences whether the other kidney is healthy. If it remains clear the likelihood is that it is. Czerny thought so, but rare cases have happened where it was not in spite of clear urine. Thus Gersuny, in Vienna, lost a case where the urine was entirely clear and at post mortem the other kidney showed only one healthy calix and the rest was infiltrated with pus. Nephrotomy alone will cure some cases by long drainage without removal of the ureteral stricture. Thus I have cured a case some twenty years ago, and the woman is living today in full health. The drainage was kept up for about a year before the fistula closed. There was a stone, but that did not constitute the hindrance for the escape of pus. Dr. Banta saw the operation. Another became pregnant and gave birth to a child before the fistula closed, and it may be open today. My paper would grow too long if I should dilate any further upon the merits of nephrotomy against nephrectomy in pyelitis, though we will have some hints concerning the question.

*Hydronephrosis.*—There are two forms: the persisting and intermittent. The first is almost always bilateral and congenital, while the latter is mostly unilateral. Whether we are helpless with the congenital remains to be seen. Landau maintains that the intermittent form is due to floating kidney and is curable by nephropexy. The hydronephrosis persistens von Kahlden considers the analogue of the ovarian kystoma, developed as a myxofibroadenoma. It becomes difficult to judge what good drainage could do here. Ewald joins this opinion with others and therefore it should not be called hydronephrosis, but cystic degeneration.

*Cystic Degeneration.*—Only two cases were diagnosticated during life and controlled post mortem: one by Dugust, one by Verneuil. The widest known seems to be the case of Leichtenstern, where one kidney weighed 1,505, the other 1,350 grams. Landau's opinion of the nature of hydronephrosis is only sometimes correct. In other cases other impediments for the escape of urine exist. Kuster had a case where, two years before, a fistula was established which would not close. He laid bare the ureter, found a stricture two and a half centimeters below the pelvis; extirpating the stricture, stitched the ureter into the pelvis and the fistula closed four months later. The urine became acid, but remained murky and albuminous.

Alsberg cured a case by using bougies through the fistula. Landau cured one case out of four in the same manner.

*Tuberculosis.*—If it is primary and readily recognised by the bacilli in the urine it gives a chance for extirpation. Israel had once seemingly lasting success by partial resection of one-third of a tuberculous kidney. I consider such proceeding dangerous, because the disease is very apt to reappear. Curettage although has been tried, but I could not find a lasting result in the literature.

Gluck says that among three or four cases in fullgrown people tuberculosis renum is duplex and in every second case in children.

*Tumors*—monocysts and echinococcus.—The latter is easy to cure by simple drainage, but hard to recognise. Berand saw only once echinococcus in both kidneys among sixty-four cases. We must not forget Spencer Wells's method of diagnosis by blowing air into the colon. Tumors can only find a cure by extirpation. I shall give statistics later on. One-half of all carcinomas in children are carcinoma of the kidney.

*Floating Kidney.*—In 1882 there were floating kidneys still extirpated and a total number of seventy-six was known. Martin alone had eight at that time, of which three died. What a difference since Hahn introduced nephropexy, with which operation he lost only one from the first fifty-six cases.

Some writers of late begin to consider operating on floating kidneys futile, as they consider it a part only of enteroptosis with rare exceptions. (Einhorn, *Medical Record*, 1898, Vol. LIV., No. 7.) Tuffier and Israel incline that way in the debate at Moscow, 1897. I cannot second these opinions in cases where localised complaint exists. Of course, we see numerous cases which we merely accidentally discover. They need no operation. Müller-Warnek's theory of duodenal compression and sequential ectasis ventriculi. Ebstein had some ninety-one cases ; sixty-five on the right side.

*Ureteritis.*—As a disease *per se* is very rare ; has been recognised only lately. As a secondary condition, it was long ago known. Israel made a nephrectomy in a case in 1893, after he had drained the kidney for two (!) days in vain. He seems not to have thought of a ureteral bougie or catheter *à demeure* at that time. Virchow says that distoma produces ureteritis in animals. In Eygpt and Abyssinia distoma hematobium is a frequent cause of hematuria and can be recognised by its characteristic eggs (with a thorn) in the urine. Filaria Bancrofti can cause the same symptoms. I do not believe that ureteritis alone will give cause in the future for nephrectomy. Bougie-dilatation and medication from above or below will suffice.

*Nephralgia.*—This is a very dark affection today. There are a number of cases in the literature where an exploratory incision with and without nephrotomy revealed nothing and yet the pain subsided. Senator attempted the explanation that the surgical procedure necessary severed some nerves and so cured the pain. I rather think it is nerve stretching in lifting the kidney. I had one case which remained well for nineteen months and then developed (duplex) tuberculosis. Dr. Carpenter found the bacilli in the urine. I abstained from attacking the kidney again because the other kidney became affected, also the lungs.

*Statistics.*—Nephrectomy is by no means child's play and this is not only because the kidney is a vital organ, but because the other suffers well-nigh invariably right after the operation. The amount of urine during the first week is decidedly diminished ; the urine often albuminous, even in those cases where the surgical kidney has long ago ceased to manufacture urine. The cause is not clear. Israel thinks it is a reflex anuria, although he saved some cases—40 per cent.—during anuro-uremic fits just by operating. Senger thinks our antiseptics being absorbed irritate the other kidney, which has to do overwork. Some accuse the chloroform.

Gross's statistics show, in 1885, 44 per cent. mortality in 233 cases of nephrectomy. Czerny had, later, the same. For sarcoma and carcinoma Gross has with laparatomy 64.8 per cent., with lumbar incision, 45.4 per cent. Only six lived over one year. One remained well for five years. With children the results are still worse. Twenty-one hydronephroses gave with transabdominal excision in seventeen cases, 41 per cent. of deaths ; four with lumbar excision, 25 per cent. of deaths. Gross has 23 per cent. deaths in ninety-three nephrotomies and twenty-one of the ninety-one surviving retained a fistula. Of those twelve were afterward nephrectomised and only one died, thus only 9.3 per cent. of death, a far better rate than in primary nephrectomy. Israel had in nineteen nephrectomies 21 per cent. of deaths. Kuster, in Marburg, has in tumors, 263 cases, 41 per cent. of deaths, but only nine remained free for three years, 3.4 per cent. Israel from his own twenty-four cases of tumor has 25 per cent. surviving three years. He removes the fatty capsula and all glands he can reach. This seems the latest advance. The next will be with the common pyelitis without obstruction.

In surgical procedures of lumbar and abdominal incisions, the latter are more dangerous.

The line of incision I prefer is starting from the point where the sacrolumbalis crosses the twelfth rib, near the spina anterior superior, which if necessary can be prolonged until near Poupart's ligament, about one inch above it.

Resection of the twelfth and even the eleventh rib has been abandoned, because this incision opens the pleura. Yet I think that if the rib is only resected and the incision remains superficial the tissues will give without the pleural sac being opened. The abdominal incision carries a danger as it severs bloodvessels of the colon, and as these have poor anastomoses they can lead to necrosis of colon. It is remarkable how many are recorded which have been lost by ileus. In large tumors, of course, the abdominal incision has the advantage that the renal vessels can be secured before enucleation is begun. If the diagnosis per abdomen can be secured and the vessels too, I would close the abdomen and proceed to lumbar enucleation. Strictures of the ureter, Fenger prefers to slit lengthwise and reunite by suturing the apex of the incision to its lower end, like Mikulicz in pyloroplasty and this is correct in short strictures, but Van Hook's proceeding remains acceptable; yet in long ones, if bougies do not cure, nothing is left but nephrectomy. Nephrotomy in distended pelves is simple, but the future operation in simple pyelitis without distension will have to go through the center (dorsum) of the kidney. In cases which reach us in a state of dangerous exhaustion, a *troicart à demeure* for a week or so will tide a pyelonephritic case (with distension) over till the patient rallies sufficiently. In metastatic multiple abscesses we may do as Howard Lilienthal did successfully twice—nephrotomy with curettage.

884 Main Street.

